# A Schema-Activation Approach to Failure and Success in Self-Control

**DOI:** 10.3389/fpsyg.2020.02256

**Published:** 2020-09-15

**Authors:** Alex Bertrams

**Affiliations:** Educational Psychology Lab, Institute of Educational Science, University of Bern, Bern, Switzerland

**Keywords:** ego depletion, energy, fatigue, schema, self-control, self-regulation, vitality, willpower

## Abstract

Numerous studies confirm the so-called ego depletion effect (i.e., self-control is impaired after an initial unrelated self-control task). During recent years, the criticism on this limited-resource approach to willpower has increased, and alternative models have been developed. I argue that the existing models cannot explain the variety of results found in the ego depletion literature and introduce the *schema model of self-control*. Referring to related schema conceptions (i.e., illness schemas and emotion schemas), I posit that the processes that cause ego depletion effects occur around the activation of the fatigue/decreased vitality schema. This schema becomes activated via the registration of behavioral and physiological changes related to exercising self-control. The activation of the schema should instigate the motivation to conserve energy and, therefore, cause reduced effort and decreased performance in a subsequent self-control task. Moderator variables (e.g., energy supply) should influence the (non)activation of the fatigue/decreased vitality schema or its consequences.

## Introduction

There is much consensus in the literature that self-control is an important ingredient for human self-regulation, which allows people to achieve long-term goals, such as academic success or health (e.g., Tangney et al., 2004; Crescioni et al., 2011; Duckworth and Seligman, 2017), and avoid unethical behaviors (e.g., Li et al., 2020; Ming et al., 2020). Self-control can be defined as restraining or overriding a response, thereby making a different response possible (Baumeister et al., 2007). For example, suppressing the impulse to eat unhealthy snacks and replacing it with the response to not consume them is considered an act of self-control (Vohs and Heatherton, 2000). Given the relevance of self-control for adjustment in life, it is important to understand how it works – and why it fails from time to time.

The aim of the present work is to introduce a new process model that may explain situational self-control failure – in particular the so-called ego depletion effect (Baumeister et al., 2018) – more comprehensively than other theoretical accounts. In brief, I assume that the previously demonstrated detrimental effect of a first self-control demand on subsequent self-control performance is mediated via the activation of a fatigue/decreased vitality schema. In my new model, it is not necessary that the fatigue or decreased vitality is consciously perceived; according to the proposed mechanism, even preconscious fatigue can be effective in this regard.

## Explanations of Self-Control Failure

The notion that human self-control is based on a limited resource or willpower may be one of the most controversial concepts in psychology today. The strength model of self-control states that after an initial act of self-control (i.e., altering a predominant response), subsequent self-control is more likely to fail as the limited self-control resource is temporarily depleted (Muraven and Baumeister, 2000; Baumeister et al., 2007, 2018). This state of reduced self-control strength, energy, or willpower has been termed ego depletion (Baumeister et al., 1998). Typically, ego depletion has been empirically demonstrated by applying a sequential-task experimental paradigm (Baumeister and Vohs, 2016), which is as follows. While some randomly assigned participants first complete a self-control-demanding task for several minutes, the control group does something superficially similar but without or less need to exert self-control. Afterward, all participants work on another self-control task as a dependent measure of momentary self-control capacity. Less self-control performance, compared to the control group, in the second task for participants who deliberately regulated their behaviors in the first task has been considered as temporary depletion of limited self-control energy. (This observed pattern will in the following be termed the ego depletion effect). The results of hundreds of single studies (Cunningham and Baumeister, 2016) and a meta-analysis (Hagger et al., 2010) have supported the existence of the ego depletion effect (for a critical review, see Friese et al., 2019).

However, there are some ambiguities regarding the strength model that have so far been insufficiently resolved. First, an attempt to replicate the ego depletion effect across 23 labs revealed a null effect on average (Hagger et al., 2016), whereas a more recent replication study across 12 labs did find the ego depletion effect (Dang et al., 2020). Moreover, skepticism and criticism on methodological and conceptual issues have been expressed (Carter et al., 2015; Lee et al., 2016; Lurquin and Miyake, 2017; Wolff et al., 2018; Friese et al., 2019). One major critical point is the nature of the limited energy resource. The idea that glucose directly underlies self-control (i.e., the degree of available glucose equals willpower) has turned out to be incorrect or at least an oversimplification (Kurzban, 2010; Beedie and Lane, 2012; Baumeister and Vohs, 2016; Vadillo et al., 2016). Another challenge for the strength model is that variables – such as the motivation to perform well (Muraven and Slessareva, 2003), positive emotions (Tice et al., 2007), or an implicit theory of non-limited willpower (Job et al., 2010) – can annul the ego depletion effect (Milyavskaya and Inzlicht, 2018). So the question becomes which mechanism allows a limited resource to function this way?

Recently, Baumeister, and Vohs (2016) extended the strength model to determine the nature of the limited resource. Picking up the explanations of Evans et al. (2016), they discussed the existence of a central governor in the brain. This governor is assumed to analyze the use of energy, and, consequently, it initiates changes in the allocation of bodily energy in order to conserve energy, thereby causing the observed ego depletion effects. However, the central governor is vague and seems to involve a problematic homunculus conception (Inzlicht and Marcora, 2016; Vandierendonck, 2016), that is, a mystical being interpreting authority within the brain. Still, I agree with the idea that there is a central mechanism for integrating information and activating related changes within the person, which can lead under certain circumstances to ego depletion effects.

Some authors have completely refused the notion of a limited resource for self-control and have explained ego depletion effects through alternative conceptions. Inzlicht et al. (2014) proposed that ego depletion is actually a motivation shift from what is externally required (have-to goals) to what is enjoyable (want-to goals). They also added respective changes in emotion to their model. In contrast, Kurzban et al. (2013) considered the observed patterns in ego depletion studies as a result of participants’ cost–benefit analysis in the lab. According to them, ego depletion effects occur only because people realize that there are better things to do with their time than persisting any longer on the assigned experimental task. Job et al. (2010) argued that ego depletion effects appear because people in the cultures where the investigations took place often have the implicit theory that willpower is limited (though it is actually not). Therefore, participants act in a corresponding manner; they reduce their self-control efforts after they have already engaged in self-control in a preceding task.

The theoretical explanations of Kurzban et al. (2013) and Inzlicht et al. (2014) are hardly compatible with the research that shows ego depletion interacts with anxiety in predicting attention-based performance. There is cumulating evidence that anxious people underperform when they are ego-depleted compared to not being depleted (e.g., Englert and Bertrams, 2012; Bertrams et al., 2013). It would not be rewarding, enjoyable, or beneficial for them to underperform and embarrass themselves; rather, they would want to perform as well as possible. Or as Baumeister and Vohs (2016) put it: “A shift from “have-to” to “want-to” is hardly beneficial if the result is the intrusion of anxious worries into one’s mind while taking an exam” (p. 99). Thus, the alternative models of Kurzban et al. (2013) and Inzlicht et al. (2014) are only applicable to a restricted range of self-control failures. Moreover, these two models do not explain why people in some studies become less susceptible to ego depletion when they regularly engage in effortful self-control (Baumeister et al., 2006; Friese et al., 2017). I am much more in favor of Job et al.’s (2010) argument that people have implicit theories about the nature of willpower and act according to them. This model is compatible with many of the existing findings on ego depletion; however, it does not directly and systematically integrate the various findings on ego depletion. Instead, Job et al.’s (2010) approach refers primarily to one particular moderator of the ego depletion effect. That focusing on a single variable is not sufficient to explain the phenomenon of ego depletion is indicated by a recent preregistered study that did not replicate Job et al.’s (2010) key findings (Carruth et al., 2018).

One further shortcoming of all the aforementioned models is that they do not explain the vicarious depletion effect (Ackerman et al., 2009; Englert and Bertrams, 2014) and the imaginary depletion effect (Graham et al., 2014; Macrae et al., 2014). The vicarious depletion effect means that taking the perspective of a fictive person who engages in effortful self-control (i.e., without actually engaging in self-control oneself) causes a decrease in subsequent self-control. The imaginary depletion effect means that people show the ego depletion effect after imagining that they control themselves without actually engaging in behavioral self-control. Since no self-control was carried out, it is hard to say what howsoever natured limited resource for self-control could have been taxed, even though the same effect of exerting self-control on subsequent self-control emerged. Likewise, neither motivational shifts nor an implicit theory of exhaustible willpower alone is sufficient to explain the vicarious and imaginary depletion effects. These theories lack the actual mechanism that leads from the pure idea of exercised self-control to real subsequent failures of self-control.

Another unsolved point is the role of fatigue in the ego depletion phenomenon. Recent considerations on fatigue and ego depletion (Wright, 2014; Wright et al., 2019) miss the crucial point. In this work, the same problem exists as with the strength model of self-control, because it does not become clear what the fatigue actually is. Again, a vague resource – reduced by an unknown process – is assumed (Wright, 2014). In their meta-analysis, Hagger et al. (2010) found a significant relationship between self-control and perceived fatigue. This led the authors to suggest that “fatigue is likely to be implicated in the ego-depletion effect” (p. 497) and that fatigue may be a mediator of the effect (see also the early work of Muraven et al., 1998). However, they also note that only a few studies have investigated the assumed mediation. In addition, beyond the general meta-analytical relationship, the findings are somewhat inconsistent at the level of individual studies. There are some studies that have demonstrated an ego depletion effect on behavior without a corresponding effect on self-reported fatigue (e.g., Bray et al., 2012; McEwan et al., 2013). In alternative theories to the strength model, fatigue is understood as an affective signal that initiates a motivational change, which in turn leads to the reduction of self-controlled behavior (Kurzban et al., 2013; Inzlicht et al., 2014). Basically, I agree with this view. However, to understand ego depletion, an explanation is also needed as to why the effect of exerting self-control on perceived fatigue did not show up consistently, and why even without an effect on perceived fatigue behavioral ego depletion effects were found. The possibility that even preconscious fatigue can mediate the ego depletion effect may be a key to resolving the inconsistent findings.

**FIGURE 1:**
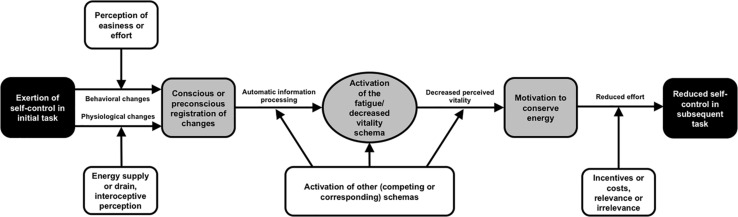
The schema model of self-control to explain the ego depletion effect. Black boxes: the observable behavior in ego depletion studies. Gray boxes and horizontal arrows: the mediating processes within the individual. White boxes: moderating variables.

In sum, there is no theoretical model at present that sufficiently and comprehensively addresses the mechanism underlying the ego depletion effect, including an explanation of the role of fatigue and the null effects found in ego depletion research. In the past, many of the found moderators of the ego depletion effect (that might explain some null findings) were neglected in developing theories on ego depletion. Therefore, I present a new model that may overcome these drawbacks. The core of the new model is the level of activation of the fatigue/decreased vitality schema. This schema is centered within several psychological and physiological processes (see Figure 1). Next, I introduce the model and cite supportive evidence from previous ego depletion research. I begin with the nature of the fatigue/decreased vitality schema and then continue with the antecedents that influence the activation of this schema. I elaborate on the activated schema and then describe the assumed consequences of the activated schema. The entire course – from the antecedents of schema activation to its consequences – involves various mediating and moderating processes, suggested by the current empirical state of the ego depletion literature.

## The Schema Model of Self-Control

### The Fatigue/Decreased Vitality Schema

Central to the schema model of self-control is a fatigue/decreased vitality schema (see the middle of Figure 1), which is activated by certain kinds of perceptions and can potentially cause motivational and behavioral changes in a given situation. The use of the term schema is not consistent across the psychological literature. I will refer to schemas as cognitive structures that contain not only facts of knowledge but also affective and motivational elements that guide people’s experience and behavior. Such conceptions of schemas can be found, for instance, in theoretical work about the nature of emotion and affect, which is a relevant domain for the present work. Izard proposed emotion schemas that “are fundamentally motivational, and … typically represent explicit or implicit affective-cognitive plans for thought and action” (2011, p. 371). According to Leventhal and Scherer, emotional schemas “are conceptualized as memories of emotional experiences: They are concrete representations in memory of specific perceptual, motor (expressive, approach-avoidance tendencies and autonomic reactions), and subjective feelings” (1987, p. 10). A different line of relevant research addresses schemas about specific illnesses (Henderson et al., 2009; Orbell and Henderson, 2016). Such schemas involve mental representations of feeling and behavior in a specific state of the self (i.e., during being ill) and strategies about how to cope with it. The term schema, as it is used here, is related to the literature on grounded cognition. According to these theories, conceptual knowledge is intertwined with the brain’s modal systems for perception, action, and introspection (Barsalou, 2008; Meier et al., 2012). Therefore, a schema can be activated by perceptions from inside and outside of the individual, which increases the accessibility of specific feelings and behavioral tendencies.

In the present work, fatigue is considered to be the opposite of vitality, lying on the contrary pole of the same one-dimensional continuum (Ware and Sherbourne, 1992; cf. Bray et al., 2012; Deng et al., 2015). As a default (i.e., not after demanding work, without being depressed, and so on), people would not feel fatigued; rather, they would feel as if they possessed some energy (i.e., feeling vital to some degree). Ryan and Frederick (1997) describe vitality as the “experience of having positive energy available to or within the regulatory control of one’s self” (p. 530). According to Martela et al. (2016), vitality supports volitional activity and performance. As “self-perceptions of available energy are generally predictive of persistence, performance, and relevant health outcomes” (Martela et al., 2016, p. 69), perceived vitality can be considered to mediate success and failure in self-control (Muraven et al., 2008; Ryan and Deci, 2008). It is noteworthy that, from a psychological perspective, vitality is a self-perception that does not necessarily correspond directly to objective physical measures, such as the expenditure of caloric energy (Martela et al., 2016). As I consider fatigue to be decreased vitality, fatigue is a perceived loss of the available energy for the self (see Ackerman, 2011 for different uses of the term fatigue).

Some results from priming research suggest that fatigue/decreased vitality may be mentally represented as a schema that can be activated by related stimuli. Fatigue and vitality are emotional variables (Noakes, 2012; Martinent et al., 2018); emotions can be conceptualized as schemas (Leventhal and Scherer, 1987; Izard, 2007, 2011), and as schemas, different emotional variables have been successfully primed (e.g., Neumann and Lozo, 2012). Therefore, one can assume that even fatigue/decreased vitality exists as a schema. In several experiments, after a subliminal priming with a fatigue-involving concept (the flu), healthy people showed elevated signs of physical and mental fatigue (e.g., decreased walking speed, impaired learning and recalling; Orbell and Henderson, 2016). Shalev (2014) found in a series of experiments that people primed with thirst (a fatigued-related bodily state) had reduced perceived momentary vitality and had lower persistence in a frustrating task (a measure that has frequently been used to assess ego depletion; e.g., Baumeister et al., 1998). These studies imply that reductions in vitality are conceptually ingrained in the long-term memory and can be activated by associated schemas, such as the flu or thirst. More directly, and especially important for the present work, Schlinkert and Koole (2018) found lowered levels of self-reported bodily vitality after a supraliminal prime of demand, which corresponds to the view that personal energy estimations can be primed (Klatzky and Creswell, 2014). Overall, there are some findings that directly or indirectly support the existence of a mental schema of fatigue/decreased vitality. Further findings (e.g., on vicarious and imaginary depletion), which I discuss later, also correspond with this schema account.

Fatigue and vitality are frequently measured by self-reports (e.g., Ryan and Frederick, 1997; Baumeister et al., 1998), which means that these perceptions are considered in such a way that they can come to consciousness. However, research suggests that the vitality or fatigue perception can also be preconscious or implicit. As mentioned above, recent studies have shown that the subliminal priming of a fatigue-involving disease can cause fatigue-related consequences (e.g., slow movement and cognitive decrements); in the same study, conscious self-reports of fatigue remained unaffected (Orbell and Henderson, 2016). These results correspond to the fact that some studies found an ego depletion effect on behavior without a respective effect on self-reported fatigue (e.g., Bray et al., 2012; McEwan et al., 2013). Moreover, affect and emotions can be implicitly activated and can operate on a preconscious level (Quirin et al., 2009; Neumann and Lozo, 2012). As vitality and fatigue can be considered emotional variables (e.g., Noakes, 2012), it is plausible to assume that vitality and fatigue can also be preconscious. As with other mental representations of emotional experience, preconscious fatigue/decreased vitality may pervade the threshold to consciousness at some point if it becomes intense enough (cf. Li et al., 2008; Quirin et al., 2009).

In sum, there is a theoretical and empirical (but still expandable) base to propose a fatigue/decreased vitality schema. In the next section, I argue that exerting self-control elicits psychological and physiological processes that activate this schema. Moreover, I point out how different moderators can influence these processes and, therefore, the degree of schema activation.

### Antecedents of the Activation of the Fatigue/Decreased Vitality Schema

Now, I outline the path from exerting self-control in a first task toward the activation or non-activation of the fatigue/decreased vitality schema (see the left half in Figure 1). Ego depletion experiments typically start with an initial task requiring self-control in the experimental condition(s) and no, or relatively little, self-control in the control condition(s). According to the schema model account, exerting self-control in the initial task releases a stronger activation of the fatigue/decreased vitality schema in the experimental relative to the control condition. There should be at least two processes that mediate this activation. These processes are in line with the conceptualization of emotion schemas, which are released by various ways, such as by appraisal processes and thoughts as well as by physiological changes (Izard, 2009).

The first way is to register the changes in one’s own behavior between the time directly previous to and during the self-control exertion. From doing something relatively easy just prior to beginning the self-control task (e.g., reading an instruction), the behavior is altered to doing something perceived as more effortful. Typically, self-control tasks and exerting self-control are perceived as being effortful as indicated by the manipulation checks in numerous ego depletion experiments (see Hagger et al., 2010). This perception may be frequently explicit and conscious so that people can report it on the manipulation check items. However, people may not always reflect on their perceptions of effort and difficulty; thus, these experiences can be also implicit and preconscious, but they still can influence behavior. Such subliminal perceptions would explain why in some studies an ego depletion effect on behavior was found, while self-reports did not indicate a corresponding effect on perceived effort or fatigue (e.g., McEwan et al., 2013).

As shown in Figure 1, the (preconscious or conscious) perception of easiness or effort during the initial self-control task should moderate the mediational effect of behavioral changes. If individuals experience working a task as easy and requiring little effort, there is actually no change from an easy to an effortful behavior, which could be registered and then activate the fatigue/decreased vitality schema. In this case, it should not matter whether or not the behavior by definition involves self-control. Supporting evidence for the suggested moderation effect comes from the re-analyses of the large dataset of the abovementioned replication study across 23 labs (Hagger et al., 2016) by Dang (2016). Dang found that the ego depletion manipulation was not perceived as depleting on average; however, participants in the depletion condition performed significantly lower in a subsequent self-control task the more effortful they had perceived the preceding depletion task. As another moderating influence, it may be of relevance at this stage of the schema model whether one’s attention is distracted from or allocated to the experience of effort during the self-control task. Accordingly, Alberts et al. (2008) found higher self-control performance in people who simultaneously engaged in an unrelated mental task during a physical self-control task.

The second way that exerting self-control can activate the fatigue/decreased vitality schema is primarily of a physiological nature. It has been demonstrated that exerting self-control causes changes in heart and brain activity. For instance, in association with self-control effort, Segerstrom and Solberg Nes (2007) found elevated heart rate variability. Inzlicht and Gutsell (2007) found a weakened error-related negativity that originated from the dorsal anterior cingulate cortex. Moreover, Friese et al. (2013) found decreased activity in the right lateral prefrontal cortex. The central governor approach to ego depletion (Baumeister and Vohs, 2016; Evans et al., 2016) argues that the central governor allocates the available glucose within the body and generates feelings of fatigue. I agree with this approach insofar that changes in the body associated with the exertion of self-control (e.g., fluctuations in glucose) are processed as a kind of information and contribute to the ego depletion effect. In line with theories on grounded or embodied cognition (Barsalou, 2008; Meier et al., 2012), I postulate that the self-control-specific changes in internal bodily states constitute stimuli that are implicitly or even explicitly registered and activate the fatigue/decreased vitality schema. This assumption is, to some extent, supported by recent work on the psychoneuroendocrinology of motor control during exercising, which integrated the related biological brain processes, interoceptive feedback, and behavioral as well as perceived fatigue (McMorris et al., 2018).

The moderators of the physiological path to schema activation are depicted in Figure 1. In accordance with the view that the interoceptive feedback may play a role in registering physiological changes leading to schema activation, interindividual differences in interoceptive awareness skills should affect this process. For example, Häfner (2013) has shown that higher interoceptive awareness skills were associated with a stronger relationship between bodily sensations and behavior. This pattern may also apply for the ego depletion mechanism (i.e., higher interoceptive awareness skills are related to stronger ego depletion effects). Another moderator may be the actual availability, or even signs of the availability, of energy in the immediate environment. Several studies have revealed that a drink of glucose (i.e., energy for the body), in contrast to a sweetened drink without energy, counteracted the ego depletion effect (Gailliot et al., 2007; Miller et al., 2010; Denson et al., 2011). Further studies have found that rinsing the mouth with glucose without consuming and metabolizing it caused the ego depletion effect to vanish (Molden et al., 2012; Sanders et al., 2012; Hagger and Chatzisarantis, 2013). An explanation is that when receptors in the mouth notice glucose, they activate the anterior cingulate cortex and the striatum – the brain areas known for being involved in self-control (Chambers et al., 2009). Thus, there is a link between glucose and physiological changes that do not require glucose to be ingested. The registration of these physiological changes should counteract or mitigate the activation of the fatigue/decreased vitality schema as it gives interoceptive feedback that energy is available, if not in the body then at least in the close environment (cf. Levy, 2016). In contrast, everything that drains energy should facilitate the activation of the fatigue/decreased vitality schema. For instance, the high-energy bodily processes during the luteal phase of the menstrual cycle can increase the likelihood of self-control failure (premenstrual syndrome; Gailliot et al., 2010; Baumeister and Vohs, 2016). People high in interoceptive awareness skills may be particularly affected by energy supply or drain in terms of its effects on the fatigue/decreased vitality schema.

When behavioral and physiological changes specific to self-control are registered, the fatigue/decreased vitality schema becomes activated via automatic information processing. Independent of whether the changes are preconsciously or consciously registered, the released schema activation is not considered to be conscious and controlled. It seems unreasonable to assume that people usually decide deliberately to be fatigued in a given moment. Rather, the registration of changes in one’s behavior and in the body works similar to a priming that is either subliminal (in the case of preconscious registration) or supraliminal (in the case of conscious registration). Even in the case of supraliminal priming, the process of schema activation itself usually remains unconscious to the primed person. To explain how conscious registration can elicit unconscious automatic processes, recent color priming research is useful as an analogy. Participants who were exposed to the color red in the background of a title page could remember having seen this color; however, they were unaware that the color – probably due to its associations with danger and failure – had affected their processing of sensations, their avoidance motivation, and their cognitive performance (Maier et al., 2008; Elliot et al., 2009). Likewise, the registration of behavioral and physiological changes during a self-control task may prime the fatigue/decreased vitality schema outside of awareness. Next, I go into detail about the middle part in Figure 1 – that is, the activated fatigue/decreased vitality schema and the potential moderators of its activation.

### The Activated Fatigue/Decreased Vitality Schema

Some findings emphasize that an activated fatigue schema causes ego depletion. The abovementioned vicarious and imaginary depletion effects can be viewed in this context. People who only mentally took the perspective of someone exerting self-control or just imagined themselves to exert self-control showed decrements in a subsequent self-control task (Ackerman et al., 2009; Englert and Bertrams, 2014; Graham et al., 2014; Macrae et al., 2014). These participants did not actually engage themselves in an initial self-control task. These findings are consistent with the activation (priming) of a schema that incorporates the concept of a lowered capacity to perform, as is the case with the fatigue/decreased vitality schema. Re-analyzing the data of Englert and Bertrams (2014) revealed that the vicarious depletion effect was present for the effortful self-control trials (i.e., interference stimuli of the Stroop test) and was absent for the non-effort trials (i.e., control trials of the Stroop test without interference). Thus, there is evidence that the fatigue/decreased vitality schema specifically affects effortful acts, such as self-control, but not every kind of behavior. Interestingly, vicariously depleted participants in Englert and Bertrams (2014) also rated the Stroop task (i.e., the self-control performance measure after the vicarious depletion manipulation) as more difficult and exhausting than the controls. Thus, just mentally imagining exerting self-control must have changed their perception. This result can be explained by the activation of the fatigue/decreased vitality schema: the schema activation has made them more sensitive to indications of effort in order to avoid them (see next section).

Furthermore, previous findings on the moderation of the ego depletion effect point to the activation or deactivation of the fatigue/decreased vitality schema. Experiments on the counterpart of vicarious depletion, namely, vicarious restoration, have shown that the ego depletion effect vanished when participants took the perspective of someone engaging in a restorative activity (Egan et al., 2012). This finding corresponds with the schema model of self-control: restoration has been primed and activated as a schema, and, thereby, the activation of the fatigue/decreased vitality schema and its effect on self-control has been overridden. Such an overriding may even be the reason why in one study the ego depletion effect disappeared after 3 min of active relaxation (Tyler and Burns, 2008). Maybe not the relaxation itself but the higher accessibility of the idea of rest was effective. In the same way, priming people with concepts related to high self-control or vitality may overlie the activation of the fatigue/decreased vitality schema. For instance, the ego depletion effect was not observable, as compared to controls, when people were supraliminally primed with the self-control-related concept of persistence (Alberts et al., 2007). Also, autonomous choice may override ego depletion effects due to its association with perceived vitality (Muraven et al., 2008; Martela et al., 2016).

The higher the self-control demands, the higher the activation of the fatigue/decreased vitality schema should be. Despite moderating circumstances that can prevent ego depletion effects (e.g., the schematic activation of the idea that willpower is non-limited; Job et al., 2010), at some point, the schema activation should be strong enough to prevail over the competing processes. For instance, in a study by Vohs et al. (2012), the ego depletion effect appeared after extensive self-control demands, even in individuals with a buffering implicit theory of non-limited willpower. In contrast, the activation of schemas corresponding to fatigue or decreased vitality (e.g., illness, sadness) may additionally contribute to an increased activation of the fatigue/decreased vitality schema and, thus, magnify the ego depletion effect. Now, I turn to the processes that are the consequences of an activated fatigue/decreased vitality schema (right half of Figure 1).

### Consequences of the Activation of the Fatigue/Decreased Vitality Schema

The activated fatigue/decreased vitality schema should provoke the appraisal that one’s energy has just decreased to some degree (i.e., the perception of decreased vitality). Again, I assume that this appraisal can be a preconscious process, which is comparable to how, for instance, negative affect and stress can operate outside awareness (Brosschot et al., 2014). However, schema activation outside of awareness can also cause explicit estimations of reduced vitality (Shalev, 2014). In the context of ego depletion research, the conscious feeling of decreased vitality should most likely occur after a period of intense self-control effort. Evidence exists that self-reported vitality decreases from before to after a demanding self-control task (Legault et al., 2009). However, the appraisal of reduced vitality does not necessarily correspond to a real loss of energy in the body (Martela et al., 2016).

The perception of decreased vitality has been found to predict lower self-control performance (Muraven et al., 2008; Gao et al., 2014). How can this relationship be explained in the context of ego depletion effects? There has been much discussion on the role of motivation within the ego depletion process (Baumeister and Vohs, 2016). I largely agree with other ego depletion theories that the reduced motivation to expend further effort leads to decrements in subsequent self-control (Kurzban et al., 2013; Inzlicht et al., 2014). In line with this notion, a recent large-scale study has shown that the decreasing trajectory of perceived self-control capacity during the course of an achievement test (possibly an indicator of how the activation of the fatigue/decreased vitality schema became stronger) was related to the decrease in the invested test-taking effort (i.e., the engagement to achieve a good performance; Lindner et al., 2018).

Deviating from alternative theories on the ego depletion effect (Kurzban et al., 2013; Inzlicht et al., 2014), I argue that the reduced effort directly arises from the deeply ingrained motivation to conserve energy rather than indirectly via an opportunity-cost calculation or the reconsideration of one’s motivational priorities. I am thus in agreement with what has long been known as the energy conservation principle (Brehm and Self, 1989; Gendolla and Richter, 2010; Richter et al., 2016). This principle holds that “given that resources are important for survival, individuals are motivated to avoid wasting them and aim at investing only those that are required for successful task execution” (Richter et al., 2016, p. 151). Since effort is a mechanism by which energy resources are mobilized (Wright, 2014), the avoidance of effort is the direct route to conserving energy.

Decreased subjective vitality may function as information that one’s energy levels have dropped and, thereby, instigate the motivation to conserve energy. Muraven and Slessareva put it as follows:

Hence, self-control breaks down after the exertion of self-control not because individuals become unable to exert self-control or because self-control becomes more difficult but because they wish to conserve strength. This is not necessarily a conscious, deliberative process but rather something individuals do continually with very little awareness. Depletion may cause people to become more selective in whether they will exert self-control. (2003, p. 904)

Corroborating this view, Job et al. (2015a) have found higher accessibility of the concept of resting and a more extensive actual resting behavior after a self-control task as compared to after a task without self-control. This finding is very compatible with the schema model of self-control: Ingrained in the long-term memory structure, the fatigue/decreased vitality schema can be understood as a network that connects conceptually related contents, such as fatigue/decreased vitality, energy conservation, rest, and inaction. The activation of the schema means that all of these contents become accessible and influential. (Note that Job et al.’s, 2015a findings were restricted to people who believed that willpower is a limited resource; this restriction is consistent with the schema model of self-control).

Why human beings tend to conserve energy could be explained by evolutionary mechanisms (e.g., Kurzban et al., 2013). There needs to be a mechanism that stops individuals from unnecessarily wasting valuable physical energy. I assume that human beings, therefore, evolved a predisposition to acquire the fatigue/decreased vitality schema, which fulfills the function of stopping unjustified effort and, thereby, conserving energy. Due to its relevance for survival, the energy conserving mechanism can be oversensitive; hence, it may even start running in the face of demands that spend no or little real bodily energy. Such oversensitivity could be given in the case for self-control (Kurzban, 2010).

When people reduce their effort to perform a self-control task, according to the schema model, reduced self-control results. This assumption is based on early ego depletion works, according to which self-control involves effort (e.g., Muraven et al., 1998). In this respect, I assume that the relationship between effort and self-control performance is roughly linear, that is, the less effort, the less self-control should be exercised. However, depending on the self-control task, there may be a point at which further effort reduction does not lead to any further self-control reduction. So someone who still invests some effort, but it is too little, can show the same observable behavior as someone who invests no effort at all (e.g., both do not drink a bitter-tasting medicine). Similarly, an observable reduction in self-control may occur only from a certain minimum reduction of effort. Such bounds are likely to depend on the type and precision of the measures chosen for both effort and self-control.

As depicted in Figure 1, the path from the motivation to conserve energy, via reduced effort, to reduced self-control in a second self-control task (in terms of a typical ego depletion paradigm) is assumed to be moderated by motivating or demotivating conditions. Several experiments have revealed that the ego depletion effect disappeared when the second self-control task was of high relevance or rewarding (e.g., Muraven and Slessareva, 2003). If an activity is rewarding or involves the self (e.g., by personal values such as helping others), the investment of effort and energy appears more likely to be justified (Gendolla and Richter, 2010). Thus, relevance and rewards can annul the motivation to conserve energy up to a certain point. However, in demanding situations, energy conservation may eventually become a strong motivation that exceeds the motivation to help others, to earn money, and so on. In one experiment by Vohs et al. (2012), information about the ostensible relevance of the tasks to perform counteracted the ego depletion effect at the beginning of the test series (as in Muraven and Slessareva, 2003); however, the relevance information became ineffective after multiple self-control tasks. From the perspective of the schema model, the activation of the fatigue/decreased vitality schema as well as the related motivation to conserve energy gradually rose the more self-control was exerted. When they became higher than the motivation from the task relevance, the self-control performance declined. For the sake of completeness, I also assume that demotivating conditions (i.e., striking costs or irrelevance related to a self-control task) would exceptionally reduce the effort invested. However, I am not aware of a study that has directly manipulated such potentially moderating demotivating variables. After introducing the schema model of self-control, I will now point out what I think this model can explain better than other ego depletion theories.

## Explanatory Value of the Schema Model of Self-Control

The schema model can explain the ego depletion effect. Thereby, it is superior to the strength model of self-control (Baumeister et al., 2018) because it is not built on a metaphor or a homunculus conception (Inzlicht and Marcora, 2016). Instead, it is an information processing approach that incorporates physiological and affective-motivational processes. Therefore, the schema model of self-control can be straightforwardly connected to contemporary research on grounded or embodied cognition (Barsalou, 2008; Meier et al., 2012).

Some findings on the moderation of the ego depletion effect could not be optimally explained by the strength model. These include in particular the finding that motivating conditions annul the ego depletion effect (e.g., Muraven and Slessareva, 2003). This finding highlighted the question about the nature of the limited self-control resource beyond a metaphor. In the schema model of self-control, the concrete positions where possible moderators can have an impact within the information processing flow are explicitly defined. This definition takes place within the framework of a logical overall structure. Thus, the schema model can integrate and explain different findings of the ego depletion literature in a coherent way, including the findings on implicit theories of willpower (Job et al., 2010). The schema model does not need the assumption of a limited resource in order to explain the ego depletion effect via an information processing mechanism; therefore, it does not have to determine the nature of such a resource. However, the schema model is basically compatible with the notion that self-control consumes a limited resource. It just puts this question in the background and states that many people act at least as if self-control would rely on a limited resource. From the perspective of the schema model, everything that activates the fatigue/decreased vitality schema can be detrimental to subsequent self-control, which can be an imagined, an imminent, or an incurred energy loss.

As to alternative theories to the strength model (Kurzban et al., 2013; Inzlicht et al., 2014), I have criticized that they simply leave out the explanation of certain findings. As also noted by Baumeister and Vohs (2016), these theories cannot account for the fact that anxiety and ego depletion combined reliably predicted decreased attention-based performance (e.g., Englert and Bertrams, 2012; Bertrams et al., 2013). The schema model, in contrast, can explain it as follows: Since the activation of the fatigue/decreased vitality schema and the resulting processes should often take place without conscious awareness, anxious people may fail to deliberately use the effort to redirect their automatic attention from their anxiety-related worries to the actual performance task. This way, the combination of both high anxiety and ego depletion can result in pronounced performance losses (cf. Bertrams et al., 2013).

Moreover, it is still unsolved which mechanism underlies the finding that regular exertion of self-control can boost self-control stamina (Friese et al., 2017). One reason may be reduced effort avoidance (Job et al., 2015b; Friese et al., 2017). This explanation aligns with the schema model of self-control in which triggered effort avoidance is considered to cause self-control decrements. Still, it remains unsolved why less effort avoidance follows from regularly engaging in self-control exercises. The schema model provides at least two explanatory approaches. First, as a consequence of frequent self-control, one could get used and be insensitive to the self-control-related feelings and physiological signals of effort up to some point. Therefore, the threshold that these experiences are registered as changes, and thereby activate the fatigue/decreased vitality schema, would rise. Second, it is possible that persistent self-control practice leads to a cognitive association between the exertion of self-control and a schema that is competing with the fatigue/decreased vitality schema (e.g., the schema of persistence; Alberts et al., 2007). If someone exercises self-control, then the competing schema is also activated, which can override the activation of the fatigue/decreased vitality schema.

In addition, in contrast to previous ego depletion theories (Job et al., 2010; Kurzban et al., 2013; Inzlicht et al., 2014; Baumeister et al., 2018), the schema model can explain the vicarious and the imaginary depletion effect (Ackerman et al., 2009; Englert and Bertrams, 2014; Graham et al., 2014; Macrae et al., 2014). According to the schema model, self-control and activation of the fatigue/decreased vitality schema should frequently occur together. Thus, the fatigue/decreased vitality schema should be associated with the cognitive representation of effortful self-control and, therefore, can be activated by the mere imagining of self-control exertion.

Perceived fatigue is conceptualized in the schema model of self-control as schema activation. This allows to explain the inconsistent findings with regard to the mediation of the ego depletion effect by self-reported fatigue (Hagger et al., 2010; Bray et al., 2012; McEwan et al., 2013). According to the schema model, it is not mandatory for people to be aware of their fatigue and to report it accurately themselves. Instead, the fatigue and the decreased motivation to invest effort can also be preconscious. That preconscious schema activation can have measurable consequences has been shown many times. For instance, subliminal priming of the flu schema (a fatigue-involving concept) slowed down physical movement and impaired cognition (Orbell and Henderson, 2016). So far, no study has investigated the mediating effect of preconscious fatigue with regard to ego depletion; there was no theoretical basis for it. The schema model offers a theoretical approach for such research.

Finally, I would like to point out that the schema model of self-control is also in line with the ego depletion literature on controlled cognition. Schmeichel et al. (2003) showed that the ego depletion effect also applies to logical reasoning, cognitive extrapolation, and thoughtful reading comprehension. Schmeichel (2007) demonstrated that an initial self-control task was detrimental for tests of working memory span and reverse digit span. He also found that completing a working memory task requiring executive control caused lowered self-control in a subsequent task. Furthermore, several studies revealed that in the state of ego depletion, reflective thinking and decision-making are impaired and that making decisions leads to diminished self-control (Baumeister et al., 1998; Masicampo and Baumeister, 2008; Vohs et al., 2008). These cognitive processes are considered to require active control by the self. Some authors have directly argued that higher-order cognitive processes, such as intelligent thinking and decision-making, involve self-control in the form of overriding predominant responses (e.g., Schmeichel et al., 2003; Vohs et al., 2008). Therefore, a parsimonious way to interpret the mentioned findings is that the entire process illustrated in Figure 1 applies to everything that requires self-control, which includes reasoning, memory updating, and making choices. I now address the questions as to whether the schema model is falsifiable and in which directions future research can go to test the usefulness of the schema model.

## Falsifiability of the Schema Model of Self-Control and Future Research

The schema model can also be used to explain the existing null findings for the ego depletion effect (e.g., Hagger et al., 2016; Bertrams and Englert, 2019; Kühl and Bertrams, 2019). One has to point to only the various evidence-based moderators that can prevent the effect to occur. However, the potential to make sense of certain null findings also endangers the model because it could be considered as being impossible to falsify. Such an accusation could be countered by the fact that the model is precise in its process and where the moderators are located (see Figure 1). Thus, it is possible to arrange studies that take these moderating variables into account (by holding them constant, measuring them, or manipulating them).

How should one attempt to falsify the schema model of self-control? Since the model is based on some existing findings, the question is not, for instance, whether incentives counteract ego depletion effects (see Muraven and Slessareva, 2003). The novel aspect of the model is that it arranges the existing evidence around a schema and that large parts of the ego depletion literature can be better understood through the associated (often preconscious) information processing mechanism. Therefore, evidence for the existence and the role of the fatigue/decreased vitality schema should be in the focus of future studies. Some of the findings I mentioned already suggest that a schema is incorporated in the ego depletion process. However, for example, it is not assured that actual initial self-control tasks, and the mediating processes they are assumed to elicit, activate the fatigue/decreased vitality schema. The model predicts that this should be the case. Should future studies fail to demonstrate that the fatigue/decreased vitality schema exists, that it becomes activated by exerted self-control, and that its activation predicts subsequent self-control, the entire schema model of self-control would implode; that is, it would be falsified.

So, following are some new paths for future research on ego depletion. First, more direct evidence should be obtained that the fatigue/decreased vitality schema exists. Such research should particularly focus on the preconscious level. For this purpose, a suitable, reliable instrument for measuring preconscious fatigue/decreased vitality must be established. Second, it should be demonstrated that preconscious fatigue or decreased vitality mediates the effect of an initial ego depletion manipulation on subsequent self-control behavior. Third, it should be shown that the assumed moderators (see Figure 1) affect the preconscious activation (or non-activation) of the fatigue/decreased vitality schema and, as a consequence, the corresponding behavioral self-control. Fourth, it should be examined how the perception of behavioral changes and the perception of physiological changes interact as antecedents of the schema activation within the phenomenon of ego depletion. Fifth, it should be addressed whether self-control trainings change the activation of the fatigue/decreased vitality schema to explain the mechanism underlying the training effects found in various previous studies.

## Final Note

In its early days, the strength model of self-control did not receive much acceptance as it did not suit the cognitivist theorizing that was predominant at that time (Baumeister et al., 2018). Then, the strength model became well-established and prominent for years. It could appear somewhat ironic that now a cognitive explanation is introduced that should help to fill theoretical gaps in the strength model. However, the new schema model of self-control is not a pure cognitivist approach as it also integrates physiological and affective–motivational process variables. It is, therefore, a model based on modern approaches of grounded or embodied cognition (Barsalou, 2008; Meier et al., 2012). Future empirical work that continues to complement the ego depletion research with the sophisticated methods of cognitive, motivation, emotion, and biological psychology will show whether a schema can explain the variety of results more convincingly than the previous theoretical accounts.

## Author Contributions

The author confirms being the sole contributor of this work and has approved it for publication.

## Conflict of Interest

The author declares that the research was conducted in the absence of any commercial or financial relationships that could be construed as a potential conflict of interest.
